# Differential Antioxidant Compounds and Activities in Seedlings of Two Rice Cultivars Under Chilling Treatment

**DOI:** 10.3389/fpls.2021.631421

**Published:** 2021-02-26

**Authors:** Shangguang Du, Xueyong Huang, Yali Cai, Yingbin Hao, Shengrong Qiu, Lihua Liu, Meng Cui, Liping Luo

**Affiliations:** ^1^School of Life Sciences, Nanchang University, Nanchang, China; ^2^School of Life Sciences, Nanchang Normal University, Nanchang, China

**Keywords:** rice seedlings, high-performance liquid chromatography, antioxidant activity, phenolic compounds, chilling treatment

## Abstract

Variations in antioxidant compounds were examined in seedlings of two rice cultivars (Qiutianxiaoting and 93-11) exposed to low temperature (4°C) for 0, 12, 36, and 48 h. Antioxidant activity was identified by 2,2-diphenyl-1-picrylhydrazyl (DPPH) and ferric reducing antioxidant power (FRAP) assays. The concentrations of total phenols, flavonoids, chlorophyll, and anthocyanins (ACNs) were determined by spectrophotometry. In addition, high-performance liquid chromatography (HPLC) was used to reveal the changes in phenolic compound concentrations in rice seedlings under chilling treatment. Results showed that antioxidant concentrations and antioxidant activity after chilling treatment were higher in 93-11 compared to Qiutianxiaoting, reaching the highest level at 36 h chilling treatment in 93-11. Phenolic compounds in Qiutianxiaoting decreased between 12 and 36 h but then increased at 48 h, whereas the corresponding levels in 93-11 increased as chilling time increased. Moreover, 10 phenolic compounds were detected and quantified by HPLC, of which gallic acid and caffeic acid tended to only exist in 93-11, whereas rutin was observed only in Qiutianxiaoting. The results of this study could be leveraged to optimize the antioxidant potential of rice in the context of healthy food choices.

## Introduction

Rice (*Oryza sativa* L.) is the most important crop in many Asian countries and feeds more than half of the world’s population (Ito and Lacerda, 2019). According to the Food and Agriculture Organization (FAO; Gennari et al., 2019), the production of rice has continued to increase over time; however, between 2016 and 2017 there was only a 0.6 percent increase in production, possibly because of adverse environmental stresses in the main rice production areas, such as floods, persistent drought, locust disasters, and so on (Bailey-Serres et al., 2019; Fang et al., 2019; Muehe et al., 2019). In general, grain, husked rice, and rice bran are the principal edible parts (Kumarathilaka et al., 2018). The use of young rice seedlings is rarely reported (Julia et al., 2018; Muehe et al., 2019). Rice seedlings have a certain potential to be used as food as well as to make beverages and cosmetic products, and could be important for helping people to maintain healthy lifestyles (Almeida et al., 2016; Eom et al., 2019). In addition, the process of growing rice seedlings is not only eco-friendly with zero waste, but can also be automatically operated in greenhouses or cultured rooms (Tamprasit et al., 2019).

A well-known example of developing functional food from seedlings is wheatgrass (*Agropyron cristatum*; Khanthapoka et al., 2015). Since the 1980s, wheatgrass sprout has been considered as a healthy food and important dietary supplement (Ben-Arye et al., 2011; Khanthapoka et al., 2015; Deroover et al., 2020). Juice squeezed from seedlings could be consumed as a drink, and seedlings powder could be added to other foods for better consumption. Wheatgrass sprout contains many functional components, such as amino acids (Bar-Sela et al., 2015), vitamins (Gore et al., 2017), minerals, and super antioxidants (Parit et al., 2018). Clinical studies have revealed that wheatgrass sprout plays an active role in alleviating symptoms associated with allergies and asthma (Devi et al., 2019) as well as distal ulcerative colitis (Ben-Arye et al., 2002; Kiran et al., 2018).

Like wheat, rice also belongs to the Poaceae family (Ito and Lacerda, 2019). Previous studies have shown that rice sprout might be used as an alternative healthy food (Tamprasit et al., 2019). Khanthapoka et al. (2015) reported that leaves harvested at the jointing stage from colored rice grass might be developed into functional foods. The fresh grass juices of rice contain lots of bioactive components with antioxidant properties, which is attributed to phenolic acids, monomeric anthocyanin, and β-carotene among others (Khanthapoka et al., 2015). The rice variety “Kum Doisaket” has the highest level of anthocyanins (ACNs), which leads to the most effective antioxidant activity and DNA protective effect (Khanthapoka et al., 2015). Tamprasit et al. (2019) studied the effect of harvest age on the phytochemical content of white and black rice grass (Tamprasit et al., 2019). It was revealed that black rice grass harvested at 5–7 days had the highest total phenols content (TPC) and total flavonoids content (TFC), while total ACNS were highest in white rice (Tamprasit et al., 2019). Earlier studies have reported that low temperature could increase the amount of antioxidants in rice; however, the difference between chilling-tolerant and chilling-susceptible varieties under cold treatment has rarely been studied (Khanthapoka et al., 2015). Furthermore, research into the antioxidation potential of rice grass is limited, and major active components which are beneficial to health and wellness are rarely known (Tamprasit et al., 2019).

Qiutianxiaoting (Q, chilling-tolerant) and 93-11 (Y, chilling-susceptible) are common in Asian countries, especially in China and Japan. In this study, seedlings of the two rice varieties were cultured for 15 days and exposed to chilling treatments (4°C) of different durations. The effect of chilling treatment was evaluated by the concentrations of total phenols, flavonoids, anthocyanin, and chlorophyll, as well as antioxidant activities. High-performance liquid chromatography (HPLC) was used to detect the phenolic compounds. The results of this study could be used to help improve the benefits associated with rice products, with more economic benefits for farmers in the future.

## Materials and Methods

### Chemicals

Analytical reagents were purchased from Aladdin Co. (Shanghai, China). Methanol, formic acid, and acetonitrile were in chromatographic grade for HPLC analysis. Ten phenolic acid standards (i.e., gallic acid, protocatechuic acid, chlorogenic acid, caffeic acid, *p*-coumaric acid, rutin, ferulic acid, salicylic acid, cinnamic acid, and kaempferol) were used at 98% purity or greater. Folin-Ciocalteu reagent, ethanol, and sodium carbonate (Na_2_CO_3_) were of analytical reagent purity for the total phenolic analysis. Sodium nitrite (NaNO_2_), aluminum nitrate [Al(NO_3_)_3_], and sodium hydroxide (NaOH) were used for flavonoids analysis. Potassium chloride (KCl) and sodium acetate (CH_3_COONa·3H_2_O) were used for detecting total anthocyanin.

### Plant Material and Chilling Treatment

Qiutianxiaoting (Q, *O. sativa* subsp. *japonica*) and 93-11 (Y, *O. sativa* subsp. *indica*) varieties were supplied by Nanchang Agricultural Bureau. Seeds of the two rice varieties were harvested in October 2019 from a farm where no chemical fertilizers or pesticides are used in Jiangxi province, China. Seeds of similar size and color were chosen and grown in plastic boxes (length: 10 cm; width: 7 cm and height: 10 cm). Seedlings of each variety were then cultured for 15 days in a plant incubator (Percival Scientific, Inc., United States), before being randomly divided into four groups for chilling treatment at 4 ± 1°C for 0, 12, 36, and 48 h, respectively. Methanol extracts from rice seedlings (MES) were obtained using a method based on Cui et al. (2019). In detail, 10 seedlings were ground into powder in liquid nitrogen. The powder (0.2000 g) was placed in a centrifuge tube with 70% methanol (1 ml), then ultrasonic extraction was performed at 65°C for 30 min. Samples were centrifuged at 4°C for 15 min (10,000 rpm). The supernatant was collected, and the residues were extracted again. Finally, the supernatants were pooled and made up to 2 ml, then were filtered using an organic filter (0.25 μm) membrane. The supernatant was stored at 4°C before analyses.

### Determination of DPPH Radical Scavenging Activity and FRAP

To determine the antioxidant activity of extracts from rice seedlings, we conducted assays of 2,2-diphenyl-1-picrylhydrazyl (DPPH) radical scavenging activity and ferric reducing antioxidant power (FRAP).

Methanol extracts from rice seedlings was used to determine the DPPH radical scavenging activity following the method of Mensor et al. (2001) with a slight modification. Briefly, rice seedling extracts (1 ml) were mixed with 0.1 mM DPPH (3 ml), and incubated at 25°C for 30 min. Absorbance was measured at 515 nm using a UV-vis-spectrophotometer (AJ1501003, China). The DPPH activity was calculated as follows:

DPPH activity (%) = [1-(A_1_-A_2_)/A_3_] *100

Where A_1_, A_2_, and A_3_ represent the absorbance values of 1 ml sample + 3 ml DPPH), 1 ml sample + 3 ml ethanol, and 1 ml ethanol + 3 ml DPPH, respectively.

Methanol extracts from rice seedlings was also used to measure the FRAP values using an existing method (Benzie and Strain, 1996) with minor modifications. The extract (0.5 ml) was added to 7.5 ml FRAP reagent and incubated for 30 min in darkness. The absorbance was taken at 593 nm. To estimate the FRAP values of unknown samples, grades of trolox (0.15, 0.3, 0.6, 0.9, 1.2, and 1.5 mM) were used to build the standard curve. For estimating the antioxidant capacity, the FRAP value was shown in μM trolox equivalent g^−1^ DM.

### Determination of Total Phenol, Total Flavonoid, Chlorophyll, and Anthocyanins Concentration

Total phenol concentration was determined using the Folin-Ciocalteu method with a minor modification (Butsat et al., 2009; Shao et al., 2014). In brief, 0.5 ml MES was added to Folin-Ciocalteu reagent (2 ml), vortexed for 30 s, and stewed for 5 min. Then 15% Na_2_CO_3_ solution (4 ml) was mixed in and incubated at room temperature (25°C) for 1 h in darkness. The absorbance of the mixture was detected at 765 nm using a UV-vis spectrophotometer (AJ1501003, China) in comparison with a blank. The actual TPC was calculated using a known standard curve of gallic acid.

Total flavonoid content (TFC) was measured using coloration methods with a chromogenic system of NaNO_2_-Al(NO_3_)_3_-NaOH (Meda et al., 2005). Briefly, 0.5 ml MES was mixed with 5% NaNO_2_ and vortexed for 30 s. After 5 min, 0.14 ml of 10% Al(NO_3_)_3_ was added and vortexed for 30 s. Then, 1 ml of 4% NaOH was mixed and incubated at 25°C for 15 min. The absorbance was measured spectrophotometrically against a blank at a 415 nm wavelength. The TFC content was calculated using a standard curve prepared using known contents of rutin (0.02, 0.04, 0.16, 0.32, 0.64, and 1.28 mg L^−1^).

Total chlorophyll content (TCC) was evaluated based on the method put forward by Ozkan and Bilek (2015), with a minor modification. In brief, 0.2000 g fresh shoot was ground in liquid nitrogen and extracted by 95% ethanol and then the absorbance was detected spectrophotometrically at 663 and 645 nm.

Total anthocyanin content (TAC) was determined based on the method put forward by Roland et al. (2017), with a minor modification. Briefly, 0.2000 g fresh tissue was homogenized in 5 ml methanol with 1% hydrochloric acid (v/v), then ultrasonically extracted for 30 min, and heated at 100°C for 1 h in a water bath. After cooling, the mixture was centrifuged at 4°C for 10 min, the supernatant was transferred to a centrifuge tube, and made up to 10 ml using methanol with 1% hydrochloric acid (v/v). The supernatant was used to measure the content of TAC by using the absorbance at 530 nm.

### HPLC Analysis of Phenolic Compounds in Rice Seedlings

#### Choice of Detection Wavelength

The detection wavelength for phenolic compounds in rice seedlings is mainly between 260 and 370 nm (Sabir et al., 2017). In our study, 260, 280, 320, and 370 nm were used to obtain the optimal detection wavelength of mixed reference standards (10 phenolic compounds).

#### Linearity Experiment

Mixed reference standard solutions with six concentration gradients were obtained by diluting 70% methanol, and each was detected by HPLC three times. The calibration curves of 10 phenolic compounds were constructed according to the peak area and concentration, which were set as the y-axis and x-axis (μg), respectively. The LOD was estimated by a signal-to-noise (S/N) ratio of 3:1, and the LOQ was estimated by a signal-to-noise ratio of 10:1.

#### Precision and Repeatability Test

The mixed standard solution was used to determine the precision of HPLC analysis, which was estimated by the reproducibility of the retention time and peak area. The mixed standards were tested six times in total, once every 6 h. The mixed standard solution was stored at 4°C before HPLC analysis, and sample stability and repeatability were evaluated six times, once every 12 h and once every 6 h, respectively by the detected retention time and peak area.

#### Standard Recovery Test

Rice seedlings were ground in to powder in liquid nitrogen, and accurately weighed (0.2000 g). The mixed solution of known concentration was then added. MES was detected by HPLC six times. The recovery of the mixed standard solution was calculated by the following equation.

Recovery = (the detected phenolic acid content of sample with standard ‐ the phenolic acid content of the sample) /the original phenolic acid content of standard solution) × 100%.

#### HPLC Analysis of Phenolic Compounds

The MES and mixed standard solutions were filtered with organic filters (0.25 μm), and 10 phenolic compounds were detected and quantified using HPLC-UV (Waters E2695, United States) based on the retention time and calibration curve. A Waters Symmetry C_18_ column (4.6 mm × 250 mm, 5 μm) was used in this study. Acetonitrile was used as mobile phases A, and 0.5% formic acid diluted with water (pH 2.35) was used as mobile phases B. The wavelength of UV detection was 260 nm, and the column temperature was 35°C. The flow rate and injection volume were set as 1.0 ml min^−1^ and 10 μl, respectively. The gradient elution method is shown in Table 1.

**Table 1:** Ratio of mobile phases for gradient elution.

Time/min	0	5	10	20	27	40	48	54	60	62
Mobile phase A	5	14	17	17	27	42	70	35	14	5
Mobile phase B	95	86	83	83	73	58	30	65	86	95

### Statistical Analysis

Each physiological index was calculated for three biological replicates, with each replicate consisting of 10 rice seedlings which were selected randomly. Means were calculated by Microsoft Excel (2016); SEs and statistical significance (*p* ≤ 0.05) based on Duncan’s test were determined using SPSS (Version 26.0, SPSS Inc., Chicago, United States).

## Results

### DPPH and FRAP Assays

**Figure 1:**
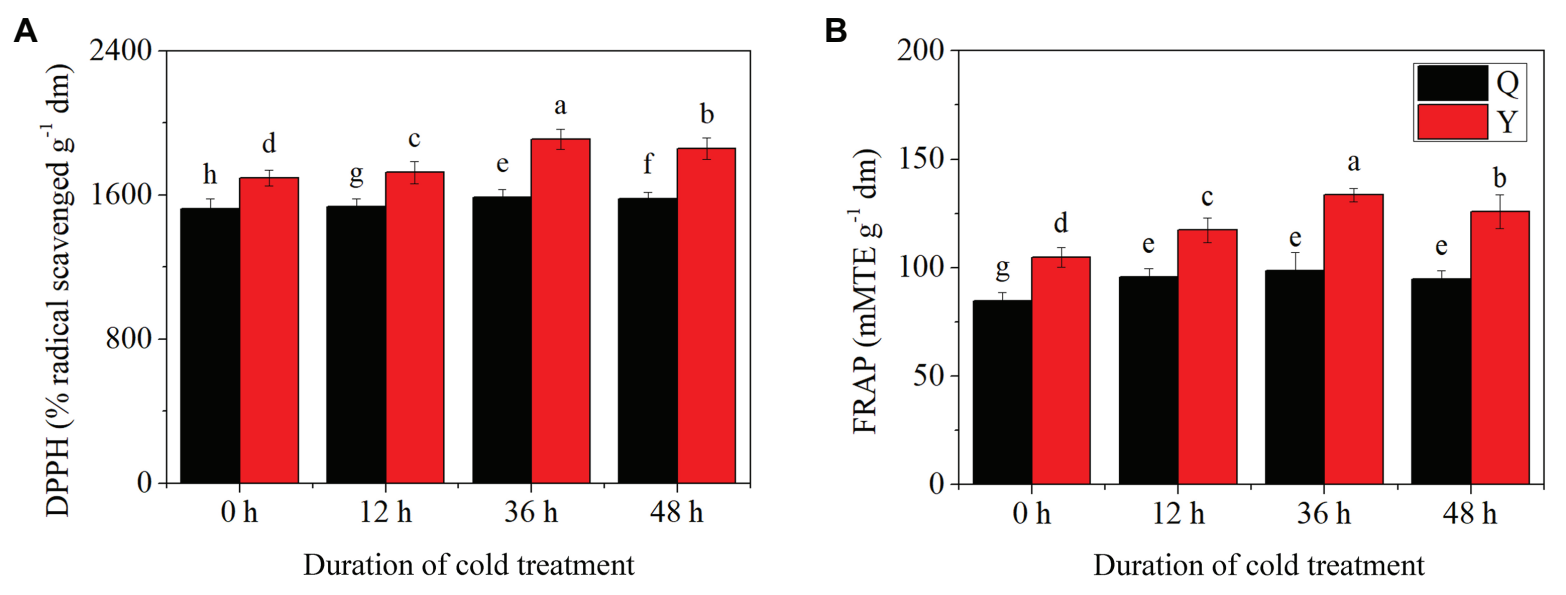
Total antioxidant activity in rice seedling extracts exposed to chilling treatments spanning different durations. (A) Antioxidant activity of 2,2-diphenyl-1-picrylhydrazyl (DPPH). (B) Antioxidant activity of ferric reducing antioxidant power (FRAP) assays. Bars represent means and SD (*n* = 3). For each group, significant mean differences (*p* < 0.05) are shown by different lowercase letters.

Results showed that significant differences existed for both DPPH radical scavenging activity and FRAP assays in the two groups after chilling treatment (Figures 1A,B). DPPH radical scavenging activity increased initially, then decreased in both groups reaching the summit at 36 h. Compared to the control (untreated group), the value of DPPH increased by 4.28 and 12.64% after 36 h chilling in Q and Y groups, respectively. The FRAP assay results exhibited a similar trend to those of DPPH after chilling treatment, increasing by 16.31 and 27.30% at 36 h in the two groups, respectively. However, there was no significant difference among chilling treated groups of Q; meanwhile the values of Y groups were greater than those of Q at the same stage under chilling treatment. In addition, the FRAP assay returned to the normal level at 48 h in group Q, while it remained at a higher level in group Y compared with the control.

### Total Phenols and Total Flavonoids Contents

**Figure 2:**
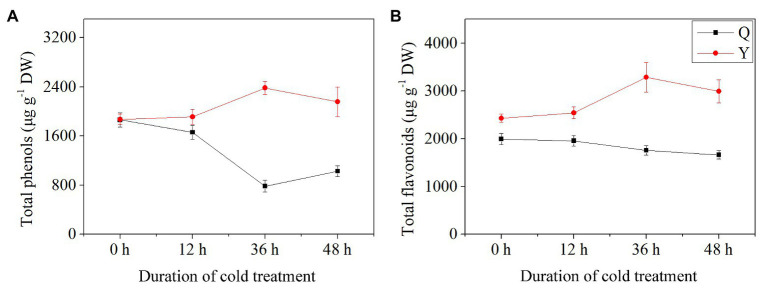
Total phenol and flavonoid contents in rice seedlings. Results are represented by means and SEs. (A) The concentrations of total phenols. (B) The concentrations of total flavonoids. DW, dry weight.

Total phenolic content and total flavonoid content exhibited differential trends between group Q and group Y after chilling treatment. TPC and TFC in Y increased initially then decreased as the duration of chilling treatment increased. The values reached the maximum at 36 h chilling in comparison with the control (untreated group), increasing by 27.33 and 35.21%, respectively (Figures 2A,B). Both TPC and TFC in group Q showed a decreasing trend compared to the control after chilling treatment, with the lowest values at 36 and 48 h, respectively (Figures 2A,B). Meanwhile, the concentrations of polyphenols including TPC and TFC reached the maximum (5671.38 μg^−1^ DW) in group Y after 36 h chilling.

### Total Chlorophyll and Anthocyanin Contents

**Figure 3:**
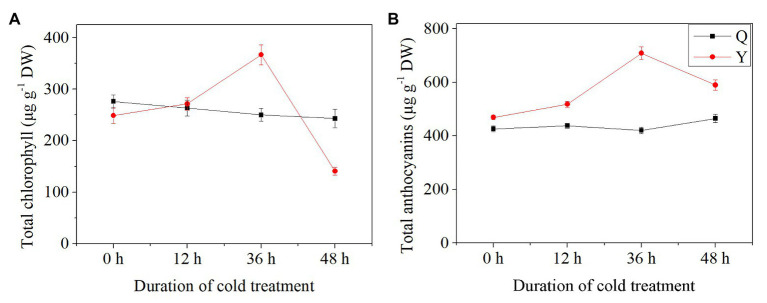
Total chlorophyll and anthocyanin contents in rice seedlings. (A) The concentrations of total chlorophyll. (B) The concentrations of total anthocyanins. Results are represented by means and SEs. DW, dry weight.

Results showed that TCC and TAC exhibited different trends between group Q and group Y. In general, the TCC of group Q decreased slightly after chilling treatment, while for group Y, TCC increased initially then decreased, reaching the highest level after chilling for 36 h (+47.52%) in comparison with the control group (Figure 3A). However, TCC decreased by 43.38% at 48 h compared with the untreated group (Figure 3A). The level of TAC showed a slight increase in group Q after chilling treatment (Figure 3B). For group Y, TAC increased after 12 h chilling and then increased by 51.19% with the highest value after 36 h (Figure 3B).

### Viability of the HPLC Method for Detection of Phenolic Compounds

**Figure 4:**
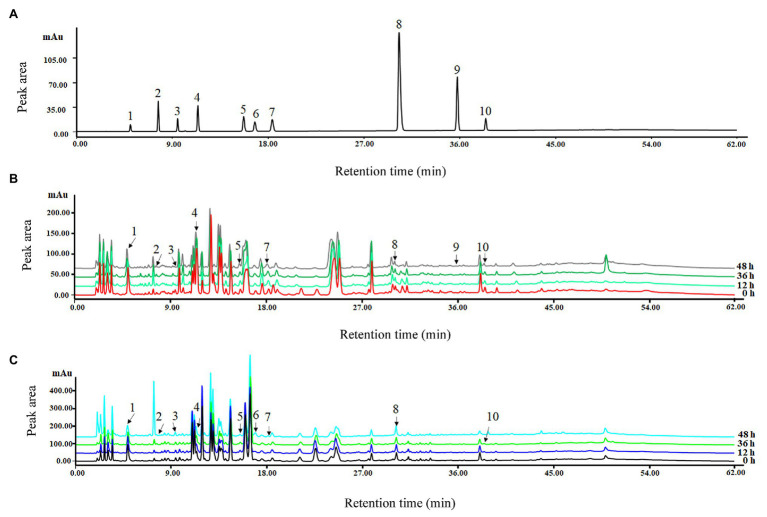
High-performance liquid chromatography (HPLC) chromatograms under 280 nm wavelength of the mixed standard **(A)** and methanol extracts for group Q **(B)** and group Y **(C)** chilled for 0, 12, 36, and 48 h. 1. gallic acid; 2. protocatechuic acid; 3. chlorogenic acid; 4. caffeic acid; 5. *p*-coumaric acid; 6. rutin; 7. ferulic acid; 8. salicylic acid; 9. cinnamic acid; and 10. kaempferol.

After comparing the signal strength, peak shapes, and separation effect, 280 nm was chosen as the best wavelength for detection (Figure 4). Linear relationship results are shown in Table 2. The correlation coefficients were between 0.9979 and 0.9999 with low LODs from 0.03 to 3.22 (μg L^−1^), which suggested that the correlation was good between the peak area and concentration of each phenolic compound (Table 2).

**Table 2:** Retention time, linear range, calibration curve, limit of detection, limit of quantification, precision, stability, repeatability, and recovery of 10 reference standards.

Standard	Retention time (min)	Calibration curve	R^2^	Linear range (mg L^−1^)	LOD (μg L^−1^)	LOQ (μg L^−1^)	Precision		Stability		Repeatability		Standard recovery rate (%)
							Peak area RSD (%)	Retention time RSD (%)	Peak area RSD (%)	Retention time RSD (%)	Peak area RSD (%)	Retention time RSD (%)	
Gallic acid	5.07	*y* = 79.451x -2.9812	0.9999	0.15–15	0.06	0.41	1.07	0.47	0.84	0.06	0.42	0.06	98.47
Protocatechuic acid	7.68	*y* = 67.397x +1.481	0.9997	0.5–50	0.71	2.64	1.98	0.61	1.25	0.03	1.51	0.14	99.51
Chlorogenic acid	9.50	*y* = 39.726x -2.541	0.9999	0.8–80	3.22	10.44	0.94	0.53	2.71	0.36	2.25	0.26	100.71
Caffeic acid	11.39	*y* = 29.151x ‐ 4.516	0.9999	0.2–20	1.24	5.43	0.82	1.12	2.28	0.02	1.54	0.13	101.34
*p*-Coumaric acid	15.66	*y* = 129.262x +3.477	0.9998	0.6–60	0.03	1.16	1.17	0.18	0.87	0.01	2.26	0.07	96.72
Rutin	16.68	*y* = 49.814x ‐ 3.431	0.9999	0.2–20	1.24	5.37	1.08	0.36	1.04	0.01	3.75	0.06	98.76
Ferulic acid	18.32	*y* = 29.385x -1.037	0.9979	0.5–10	0.49	0.74	1.41	0.79	1.41	0.01	2.53	0.02	96.45
Salicylic acid	30.25	*y* = 89.177x +2.385	0.9998	0.7–70	0.15	0.24	0.97	0.37	0.79	0.09	1.65	0.16	86.92
Cinnamic acid	33.87	*y* = 57.221x -13.713	0.9998	0.5–50	0.03	0.22	1.13	0.06	1.68	1.26	1.82	0.14	96.55
Kaempferol	38.42	*y* = 35.216x -4.172	0.9999	0.05–5	0.51	1.57	3.79	0.09	1.72	1.96	2.24	0.38	105.29

The precision, stability, and repeatability of 10 phenolic standards are also shown in Table 2, together with the relative SD (RSD) of each peak area and the corresponding retention time. The range of peak area RSDs was 0.82–3.79% in the precision test, with retention times of 0.06–1.12%, indicating that the precision of detection was sufficient in mixed standard compounds. The standard recovery rates ranged from 86.92 to 105.29%, suggesting that the processing of extraction was reliable with minor loss of phenols, and the extraction method was stable for the quantitative analysis.

Stability test results showed that peak area RSDs were between 0.79 and 2.71%, and that retention time RSDs were between 0.01 and 1.96%. These results indicate that the mixed standard of phenols was stable in terms of HPLC detection (Table 2).

The results of repeatability testing showed the range of peak RSDs was 0.42–3.75%, and retention time was 0.02–0.38%. This indicates that the repeatability was adequate during the process of detection (Table 2).

### HPLC Analysis for Phenolic Compound Contents

Ten phenolic compounds were detected and quantified in rice seedlings under different chilling treatments by using standards of phenols. In general, most phenolic compounds showed confusing trends in the two groups; whereas the total content of phenolic compounds exhibited a significant trend after chilling treatment. In detail, it decreased between 12 and 36 h and then returned to the normal level at 48 h in group Q; whereas levels in Y showed a rising trend as the chilling time increased, reaching the highest value at 36 h (Table 3).

**Table 3:** Contents of 10 phenolic compounds in methanol extracts from rice seedlings (MES) after different chilling treatments (μg g^−1^ DW).

Treatments	Gallic acid	Protocatechuic acid	Chlorogenic acid	Caffeic acid	*p*-Coumaric acid	Rutin	Ferulic acid	Salicylic acid	Cinnamic acid	Kaempferol	Total
Q0 (0 h)	80.44 ± 4.03d	nd	54.66 ± 0.46b	217.48 ± 10.20c	58.45 ± 3.2b	193.83 ± 1.60bc	111.65 ± 1.69c	20.17 ± 0.37e	nd	33.28 ± 2.30d	769.96 ± 21.88c
Q1 (12 h)	83.85 ± 0.88d	23.49 ± 1.39bc	47.72 ± 1.36c	45.65 ± 1.30d	67.55 ± 2.40ab	190.55 ± 3.10c	123.29 ± 2.63b	20.22 ± 0.16e	nd	24.27 ± 0.71e	626.59 ± 18.37d
Q2 (36 h)	63.14 ± 2.36d	27.74 ± 0.99b	46.40 ± 1.38c	44.64 ± 1.65d	65.05 ± 2.97ab	203.29 ± 3.45b	107.23 ± 2.79c	10.84 ± 0.27e	nd	nd	568.33 ± 15.86d
Q3 (48 h)	51.85 ± 2.77e	13.08 ± 5.00c	58.89 ± 4.06a	210.30 ± 31.2c	57.37 ± 5.40b	217.59 ± 17.42a	141.54 ± 12.57a	51.43 ± 3.93d	nd	nd	802.05 ± 82.35c
Y0 (0 h)	55.34 ± 4.85e	23.82 ± 8.43 b	28.77 ± 3.62d	270.00 ± 32.7b	75.11 ± 4.41a	nd	94.49 ± 9.61d	92.27 ± 11.71c	35.81 ± 6.26a	81.31 ± 8.01a	756.92 ± 89.60c
Y1 (12 h)	380.67 ± 23.12c	26.97 ± 8.47b	17.38 ± 0.51f	212.21 ± 8.35c	63.21 ± 4.17b	nd	122.08 ± 3.81bc	103.99 ± 1.35b	13.09 ± 1.34c	47.13 ± 1.02b	986.73 ± 47.71b
Y2 (36 h)	534.21 ± 31.22a	16.76 ± 3.12c	21.39 ± 2.63e	434.16 ± 30.4a	54.25 ± 6.19b	nd	101.56 ± 5.8 cd	117.23 ± 11.06a	nd	35.98 ± 1.05c	1315.54 ± 91.47a
Y3 (48 h)	454.67 ± 22.8b	28.96 ± 9.59a	16.12 ± 0.73f	189.35 ± 16.90c	64.56 ± 3.12ab	nd	122.78 ± 3.56b	112.38 ± 4.1ab	25.84 ± 1.92b	51.36 ± 2.32b	1066.02 ± 65.04b

Different lowercase letters indicate significant difference at *p* < 0.05. DW, dry weight. “nd,” not detected.

For group Q, the concentrations of gallic acid, protocatechuic acid, and *p*-coumaric acid increased initially then decreased after chilling treatment. However, the concentrations of chlorogenic acid and caffeic acid decreased first then increased. Moreover, the concentrations of rutin, ferulic acid, and salicylic acid increased from 193.83, 111.65, and 20.17 μg g^−1^ DW to 217.59, 141.54, and 51.43 μg g^−1^ DW at 48 h, respectively.

For group Y, gallic acid and caffeic acid were major phenol acids under chilling treatment, whose concentration reached the maximum value at 36 h, increasing by 865.32 and 60.80%, respectively. Levels of protocatechuic acid and kaempferol decreased first then increased after chilling treatment. Meanwhile, the levels of phenolic compounds with a lower concentration decreased and changed little at 0–48 h, namely chlorogenic acid, *p*-coumaric acid and cinnamic. Levels of ferulic acid and salicylic acid increased from 94.49 and 92.27 μg g^−1^ DW to 122.78 and 112.38 μg g^−1^ DW at 48 h, respectively.

## Discussion

Rice is a staple food in some Asian countries and it is widely distributed all over the world (Gennari et al., 2019). In general, rice can be classified into *japonica* (cold-tolerant varieties) and *indica* (cold-sensitive varieties). Phytochemical content dynamics, especially with respect to antioxidant substances, are rarely reported for these two varieties under chilling treatment (Zhu et al., 2017). It is important to increase antioxidant concentrations in order to enhance the potential value of rice. Previous reports have revealed that seedling juices and extracts from rice seedlings exhibit a certain antioxidant capacity (Gennari et al., 2019). The results of our study showed that Y (cold-sensitive variety) contained higher levels of antioxidants compared to Q (cold-tolerant variety), with the maximum value in group Y occurring after chilling for 36 h; this indicates that Y has the potential to be utilized as a healthy food choice (Figures 1A,B).

Zhang et al. (2016) reported that the content of polyphenols (phenols and flavonoids) increased in rice seedlings under low temperatures, and that phenols might act as antioxidants to scavenge free radicals generated under chilling stress. Kyriacou et al. (2019) examined 13 common microgreen species in terms of compositional variation. Results showed that phenolic composition (phenols and flavonoids) exhibited high antioxidant capacity, ranging from 691 to 5920 μg g^−1^ DW. In our study, higher total phenols and flavonoids were observed in the rice seedlings of Y after chilling treatment, and phenolic compounds reached 5671.38 μg^−1^ DW at 36 h (Figure 2A), which indicates that Y might be considered as a novel functional food with desirable properties from the perspective of healthy lifestyles. Additionally, low temperature plays an important role in plants during the growth period: it can induce the production of reactive oxygen species (ROS), which may activate phenol biosynthesis-related genes in plants (Chinnusamy et al., 2007). In this study, phenols occurred at a higher level after chilling treatment which suggests that further research exploring chilling optimization (in terms of time and temperature) for maximizing phenol content, would be valuable (Figure 2A).

Chlorophyll is widely present in plants, and it has been found in seedling juices from barley (Zhou et al., 2020). Chlorophyll has a similar structure to hemoglobin, and could be used as a substitute for it in clinical contexts (Zhou et al., 2020). Chlorophyll is also used in drinks in some Thai cultivars of rice seedlings with content between 0.07 and 0.8% solids (Wiwat and Suthaya, 2016). It has been reported that barley seedling juices have a clinically beneficial effect in terms of hemoglobin when eaten as an adjuvant over 1 year (Li et al., 2019).

Anthocyanins, obtained from botanical extracts, are a class of flavonoids which include a heterocyclic ring and two aromatic rings; these endow them with significant antioxidant potential to scavenge ROS (Banerjee et al., 2020). For this reason, ACNs might be a better choice instead of synthetic antioxidants. Moreover, some researchers have reported that ACNs can increase the stability of protein-based colloidal systems by combining with proteins through covalent and non-covalent bonds (Pham et al., 2019). ACNs could also shield lipids from oxidation, especially unstable fatty acids, by forming interfacial barriers (Elias et al., 2008). ACNs extracted from rice could be used as natural antioxidants (Yi et al., 2020).

Polyphenols including phenolic acid and flavonoids are well-known as a class of important secondary metabolites; gallic acid, *p*-coumaric acid, and vanillic acid are soluble free or conjugated in rice (D’Amato et al., 2018). In our study, polyphenols were more abundant in Y compared to Q; and gallic acid, caffeic acid, and rutin showed obvious differences between the two groups (Table 3). Gallic acid and caffeic acid were major phenols existing in group Y, while rutin was only identified in group Q (Table 4).

Gallic acid is the dominant phenolic acid in most plants and is of great importance for growth and development (Chao et al., 2018). Gallic acid is considered as a bioactive metabolite in plants with anti-oxidation, antibacterial, and anticancer properties (Chao et al., 2018). However, its antioxidant property has been of particular interest to researchers (Ahmed et al., 2018). Gallic acid is applied as a raw material to produce trimethoprim which is widely used for treating prostatitis and urinary tract infections (Fernandes and Salgado, 2015). It is also used as a preservative in food and beverages mainly because of its free radical scavenging ability (Fernandes and Salgado, 2015).

Caffeic acid is abundant in nature and exhibits anti-oxidation behavior (Khan et al., 2016). It is found in significant quantities in plants, such as rice, coffee, fruit, vegetables, and tea (Choi et al., 2017; Liu et al., 2019). In general, free radicals produced in humans can damage DNA, proteins, and cells, which lead to pathophysiological disturbances (Liu et al., 2019). Foods rich in caffeic acid exhibit antioxidant capacity with the potential to scavenge free radicals (Khan et al., 2016). Moreover, caffeic acid is widely applied as an antioxidant to promote hematopoiesis and hemostasis (Lee and Back, 2019).

The level of rutin content exhibited an obvious increasing trend in group Q but it was not detected in Y (Table 3). Rutin is extracted from natural resources such as oranges, grapes, and limes (Akanksha et al., 2016). Rutin is known as a flavonoid and considered as vitamin P, which acts as a vital bioactive compound in plants (Akanksha et al., 2016). Furthermore, previous studies have reported that rutin has an antihyperglycemic effect by preventing the small intestine from absorbing carbohydrates and stimulating beta cells to secrete insulin (Bijou-Adaze et al., 2018). In addition, some reports show that rutin, as an antioxidant, plays an important role in neurodegenerative diseases, such as Alzheimer’s disease, Parkinson’s disease, and Huntington’s disease (Bijou-Adaze et al., 2018).

## Conclusion

The concentrations of total phenols, flavonoids, chlorophyll, and anthocyanins were quantified in two varieties of rice seedlings (Q and Y groups) after exposure to chilling treatments spanning different durations. Further, antioxidant activity was measured by DPPH and FRAP assays. Under chilling treatment, the concentrations of these phytochemicals increased to a greater extent in group Y compared to group Q, with the maximum value occurring after 36 h in group Y. An effective method was also provided for the detection of 10 phenolic compounds extracted from rice seedlings.

The results show that phytochemicals such as phenols, flavonoids, chlorophyll, and anthocyanins were abundant in the seedlings from group Y. Higher antioxidant activity was also observed in Y after chilling treatment. Therefore, Y seedlings could be considered for developing functional foods or novel sources of antioxidants; meanwhile, chilling treatment can be used to increase the antioxidants in rice seedlings to optimize product efficacy.

## Data Availability Statement

The original contributions presented in the study are included in the article/supplementary material, further inquiries can be directed to the corresponding author.

## Author Contributions

SD, XH, and YC designed the research. The experiments were carried out by SD under the supervision of MC and LL. YH, SQ, and LL helped with the logistics support for the experiment. All authors have read and approved the final manuscript.

### Conflict of Interest

The authors declare that the research was conducted in the absence of any commercial or financial relationships that could be construed as a potential conflict of interest.
